# Automated detection and segmentation of pleural effusion on ultrasound images using an Attention U‐net

**DOI:** 10.1002/acm2.14231

**Published:** 2023-12-13

**Authors:** Libing Huang, Yingying Lin, Peng Cao, Xia Zou, Qian Qin, Zhanye Lin, Fengting Liang, Zhengyi Li

**Affiliations:** ^1^ Department of Ultrasound Shenzhen Second People's Hospital The First Affiliated Hospital of Shenzhen University Shenzhen China; ^2^ Shenzhen University School of Medicine Shenzhen China; ^3^ Department of Diagnostic Radiology The University of Hong Kong Hong Kong China; ^4^ Department of Ultrasound Longgang District People's Hospital of Shenzhen Shenzhen China

**Keywords:** an Attention U‐net, deep learning, pleural effusion, segmentation, ultrasound

## Abstract

**Background:**

Ultrasonic for detecting and evaluating pleural effusion is an essential part of the Extended Focused Assessment with Sonography in Trauma (E‐FAST) in emergencies. Our study aimed to develop an Artificial Intelligence (AI) diagnostic model that automatically identifies and segments pleural effusion areas on ultrasonography.

**Methods:**

An Attention U‐net and a U‐net model were used to detect and segment pleural effusion on ultrasound images of 848 subjects through fully supervised learning. Sensitivity, specificity, precision, accuracy, F1 score, the receiver operating characteristic (ROC) curve, and the area under the curve (AUC) were used to assess the model's effectiveness in classifying the data. The dice coefficient was used to evaluate the segmentation performance of the model.

**Results:**

In 10 random tests, the Attention U‐net and U‐net ’s average sensitivity of 97% demonstrated that the pleural effusion was well detectable. The Attention U‐net performed better at identifying negative images than the U‐net, which had an average specificity of 91% compared to 86% for the U‐net. Additionally, the Attention U‐net was more accurate in predicting the pleural effusion region because its average dice coefficient was 0.86 as opposed to the U‐net's average dice coefficient of 0.82.

**Conclusions:**

The Attention U‐net showed excellent performance in detecting and segmenting pleural effusion on ultrasonic images, which is expected to enhance the operation and application of E‐FAST in clinical work.

## 1 INTRODUCTION

Trauma often leads to active bleeding as a result of damage to vital organs.^1^, ^2^, ^3^ Control of bleeding after trauma is the leading preventable cause of death in injured patients.^4^ Failure to promptly identify potential bleeding is the primary cause of death among trauma patients within 24 h of admission.^4^, ^5^ In patients experiencing hemodynamic instability, there is a progressive accumulation of fluid in the chest, pericardium, and abdominal cavity.^2^ Therefore, effusion in the serous cavity is an important clinical sign to indicate parenchymal organ damage. Early detection of free effusion is helpful for the timely treatment of trauma patients and improves the survival rate and prognosis.

The Extended Focused Assessment with Sonography in Trauma (E‐FAST) exam is a common practice in the initial assessment of trauma patients.^6^ E‐FAST is a rapid ultrasound examination used to screen for pericardial, abdominal free fluid, and thoracic injuries.^2^, ^7^, ^8^, ^9^ It allows clinicians to diagnose significant trauma at the bedside quickly and without ionizing radiation.^7^ A positive E‐FAST exam has been demonstrated as a strong independent predictor for the need of surgical intervention and critical decision‐making in trauma cases.^8^ Therefore, the timely detection of free fluid with the E‐FAST exam has been shown to reduce the time to surgical intervention and improve resource utilization.^8^ One of the key components of the E‐FAST protocol is the detection and evaluation of pleural effusion. However, the diagnostic ultrasound examination in E‐FAST relies on the operator's proficiency, and correct technical execution and image interpretation are essential for achieving high diagnostic accuracy,^5^ particularly in trauma situations. Moreover, the demand for rapid assessments in emergency situations places additional challenge on sonographers. Therefore, automated solutions applied to medical images, such as Artificial intelligence (AI), can help address these limitations.^10^

Medical image analysis utilizing AI has gained widespread acceptance in the medical field.^11^ In recent years, deep learning models have been employed to address diagnostic tasks in E‐FAST in various studies.^8^, ^12^, ^13^, ^14^, ^15^ Lin et al.^14^ employed the U‐net to detect and segment ultrasonic images of ascites. Brittany E Levy et al.^12^ used four convolutional models to detect ascites separately. Cheng et al.^13^ utilized the ResNet50‐V2 to detect free fluid in Morison's pouch. Yıldız Potter et al.^8^ employed the YoloV3 algorithm to automatically detect and locate pericardial effusion. These studies mark a significant step forward in the field of emergency medicine. While these studies primarily focused on the study of pericardial and abdominal free fluid, research on the inclusion of ultrasound images of pleural effusion in deep learning is limited. To our knowledge, Tsai et al. were the only ones who trained a Reg‐STN model to detect pleural effusion in a binary manner in 2021, achieving an average accuracy of 92.4%.^16^ In our study, we aimed to further advance this research by attempting to segment and visualize pleural effusion using ultrasonic images. Automated solutions can better assist clinicians in their work, especially in emergency situations, which is the main purpose of this study.

In this study, we adopted a fully supervised learning approach to train the Attention U‐net models on ultrasound images of 848 subjects. The aim of this study was to develop an AI diagnostic model that enables prompt and accurate detection, as well as robust segmentation, of pleural effusion in ultrasound images. This model is intended to assist novice physicians, clinicians lacking ultrasonic imaging expertise, or non‐professional individuals in quickly evaluating pleural effusion through ultrasound images. We anticipate that our model will enhance the diagnostic efficacy of E‐FAST, enabling a more precise and effective diagnosis and treatment of trauma patients with active bleeding.

## 2 METHODS

### 2.1 Subjects

**FIGURE 1:**
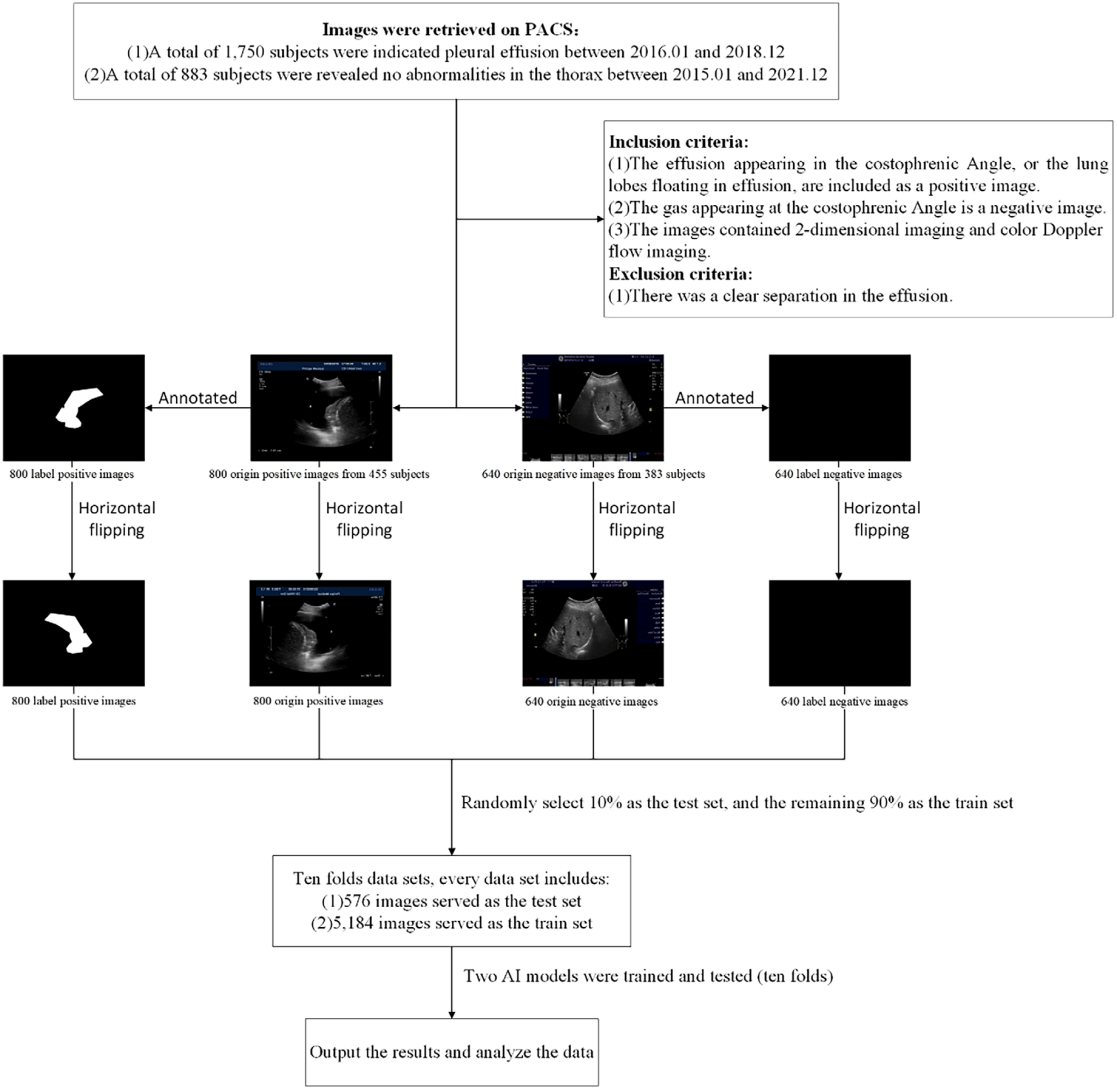
Flowchart of deep learning for pleural effusion diagnosis using ultrasonography images.

We conducted a retrospective analysis of ultrasound images retrieved from the Picture Archiving and Communication Systems (PACS) of the Ultrasound Department. Between January 2016 and December 2018, 1750 ultrasound examinations revealed pleural effusion, while between January 2015 and December 2021, 883 ultrasound examinations showed a normal chest cavity without pleural effusion. A flowchart depicting the study design is presented in Figure 1.

The inclusion criteria for the experiment are as follows: (1) The effusion appearing in the costophrenic angle (namely, both effusions build‐up and the liver or spleen can be seen on the ultrasound image), or the lung lobes floating in effusion are included as a positive image. (2) The gas appearing at the costophrenic angle (namely, both gas and the liver or spleen can be seen on the ultrasound image) is a negative image. (3) The images contained two‐dimensional imaging and color doppler flow imaging. The exclusion criteria are as follows: (1) The image was excluded if there was a clear separation in the effusion. Due to the lack of a standard section in ultrasound images and the irregular shape of pleural effusion, our study's inclusion and exclusion criteria were based on the anatomical structure of the costophrenic angle. The pleural effusion initially builds up in the pleural cavity's lowest part, the costophrenic recess. Meanwhile, the doppler principle can help us determine whether fluid flows in an ultrasound image. It can also be used to identify blood vessels and fluid in the human body.

After re‐reading the images, a total of 800 positive images (from 455 subjects) and 640 negative images (from 393 subjects) were included in the study. Two experienced sonographers evaluated all ultrasound images.

### 2.2 Data set design

As depicted in Figure 1, all ultrasound images, including positive and negative images, were manually annotated with regions of interest (ROIs) in the pleural effusion area, excluding areas obscured by acoustic shadows. The negative images do not outline any ROIs. The annotations were performed using GNU Image Manipulation Program (GIMP, version 2.10.22). Horizontal flipping was utilized to augment the dataset and retain the features of the enhanced echo behind the pleural effusion. Image Tuner (version 4.1) was used to process the flipped images. As shown in Figure 1, there are 5760 images in total. The original set includes 1440 ultrasound photos, of which there were 800 positive images and 640 negatives. All the original images were annotated to form a label set (1440 label images). Finally, the original and label set were flipped horizontally. The dataset was split into a training set and a test set, each containing two folders—one for the original ultrasonic image and the other for the corresponding label image. The test set comprised 10% of randomly selected positive and negative images, while the remaining 90% were used for the training set. The test set consisted of 576 photos, including 288 ultrasound images and 288 annotated images. The training set included a total of 5184 images, with 2592 ultrasound images and 2592 annotated images. To perform cross‐validation, ten folds of training and test sets were created, with each image appearing once in the test set.

### 2.3 Data preprocessing

The original images are provided in RGB format. First, we convert these images to grayscale. Second, we standardize the image size, fixing the picture size to 128 × 128 × 1. Third, the label picture data type is converted from uint8 to float32 and mapped to the range 0 to 1. Finally, due to the limited number of images in the data set, it is necessary to enhance the image data processing in the experiment to improve the generalization ability of the network. In this paper, the horizontal flip operation is used to expand the data set to twice the original size and increase the number of network training samples.

### 2.4 Experimental environment

In this paper, based on a split task, all models are carried out in same environment. The selected experimental models are the U‐net and the Attention U‐net. All programs were performed on an Intel(R) Xeon(R) computer with a single NVIDIA TITAN Xp GPU. The photos were processed using a Windows 10 computer and Python (version 3.6).The networks were developed in Tensorflow and Keras environments. The Adam optimizer was used to optimize the model parameters, and the learning rate was 0.0001. A combined soft dice loss and cross‐entropy loss were used as the loss function in all training processes.

### 2.5 Model

**FIGURE 2:**
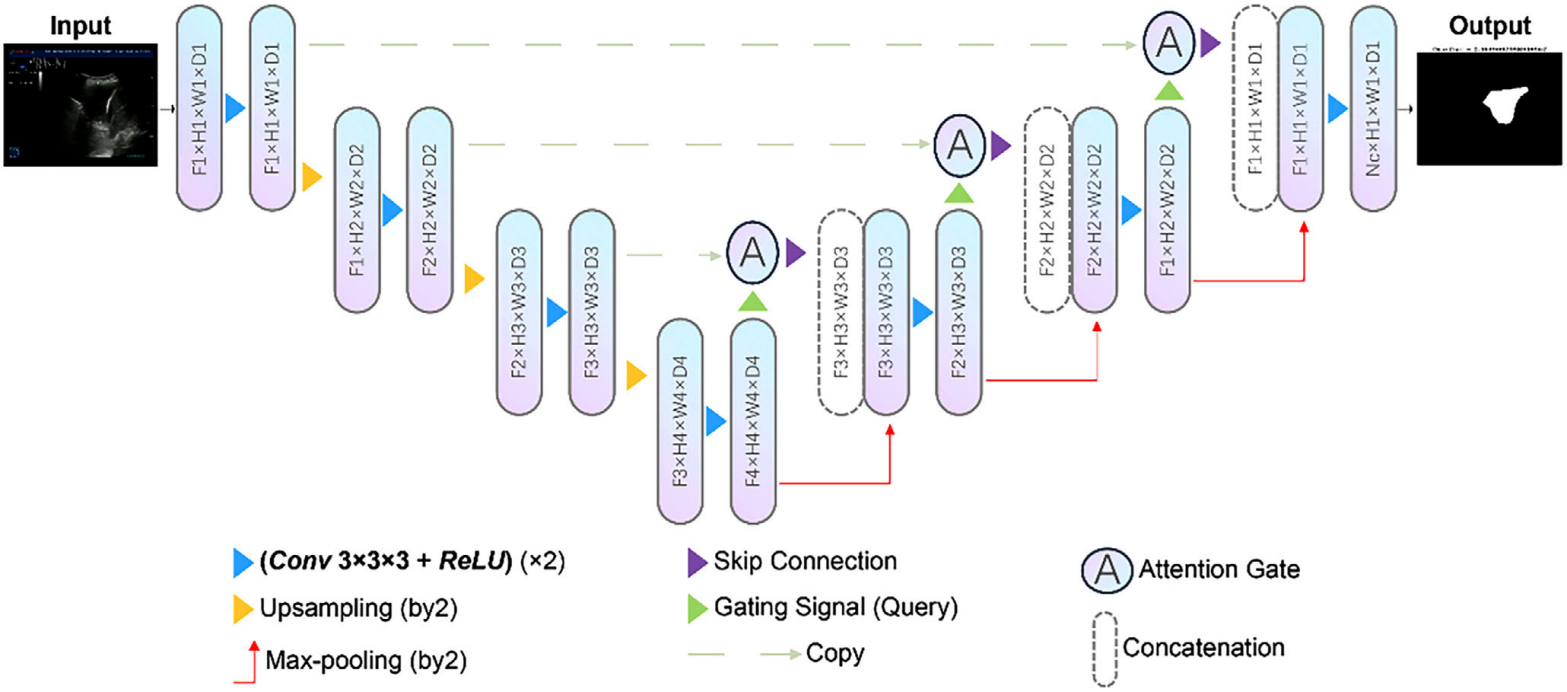
Network structure of the Attention U‐net model with the ultrasound images for pleural effusion. The remarkable feature of the Attention U‐net was the additional the AGs in between the contracting and expansion paths. AGs, attention gates.

(1) U‐net: The U‐net model, which has both contraction and expansion paths, is commonly used as the basis of biomedical image segmentation networks.^17^ Each path consists of four convolution blocks. The bottleneck section of the contracting path, located at the bottom, is fed into an up‐sampling layer on the expansion path.^17^, ^18^ The contracting path on the left is continuously stacked with convolution and maximum pooling operations, increasing the receptive field and gradually decreasing the input size at each level, while the number of output feature maps gradually increases. In this way, the features are constantly abstracted, and the correlation of spatial positions is gradually reduced. In the right expansion path, the image original size is gradually restored through up‐sampling and convolution operations. At the same time, the same level and the shallow features with strong spatial correlation are spliced and fused by using the cross‐connection. This design can achieve high accuracy semantic segmentation.^17^, ^18^ (2) Attention U‐net: The Attention U‐net is an extension of the U‐net model that utilizes attention gates (AGs) to improve prediction performance.^19^ The AGs were integrated into the U‐net architecture, as shown in Figure 2. In the Attention U‐net, the up‐sampled feature map of the decoder part and the feature map of the encoder part are used to construct the pixel weight map. This weight map is calculated by the similarity between the feature maps at the corresponding positions. Then, by using this weight graph, the feature graph of the next layer of the same layer of the under‐sampling layer is gated to obtain the weighted feature graph. This mechanism enables the network to pay more attention to and retain the encoder feature maps that are similar to the decoder feature maps, thus improving the accuracy of semantic segmentation.^20^, ^21^

### 2.6 Evaluation indices and statistical analysis

Based on the confusion matrix, we compute sensitivity, specificity, precision, accuracy, and F1 score and use these metrics to assess the model's effectiveness in classifying the data. The receiver operating characteristic (ROC) curve and the area under the curve (AUC) were also used to evaluate the model's classification performance. The closer these evaluation indicators are to 1, the better the predictions are.^22^ The *T*‐test was performed to analyze the different results between the U‐net and the Attention U‐net. Statistical significance was defined as *p*‐value < 0.05.

The dice coefficient, a measure of contour consistency for segmented lesions occupying the reference position, was used to evaluate the segmentation performance of the model. The manually annotated images were regarded as a reference and compared with the models' results.

## 3 RESULTS

### 3.1 Baseline characteristics of images

Table 1 lists the patients who have been enrolled, along with their age, gender distribution, and the ultrasound equipment type. A total of 848 subjects were extracted from the ultrasonic database (PACS). The mean age was 51.9 (±18.3) years old. A total of 446 men and 402 women were included in the study. The images were derived from 25 models of ultrasonic equipment. More than 60% of the subjects were examined using ultrasonic examination equipment of the Philips IU22, Esaote MyLabTwice, Philips Epiq7C, Philips Epiq5, and GE LogoqE9, which also provided the majority of the images used in the study.

**TABLE 1:** Patient characteristics in this study.

Characteristics	Positive (*n* = 455)	Negative (*n* = 393)	Total (*n* = 848)
**Age**	54.2 ± 18.0	49.1 ± 18.3	51.9 ± 18.3
**Sex**			
Male	264 (31.13%)	182 (21.46%)	446 (52.59%)
Female	191 (22.52%)	211 (24.88%)	402 (47.41%)
**Machine categorical**			
Philips IU22	104 (12.26%)	72 (8.49%)	176 (20.75%)
Esaote MyLabTwice	75 (8.84%)	52 (6.13%)	127 (14.98%)
Philips Epiq7C	44 (5.19%)	62 (7.31%)	106 (12.50%)
Philips Epiq5	45 (5.31%)	58 (6.84%)	103 (12.15%)
GE Vivid E9	21 (2.48%)	31 (3.66%)	52 (6.13%)
GE Logiq S8	26 (3.07%)	19 (2.24%)	45 (5.31%)
GE Logiq E9	23 (2.71%)	11 (1.30%)	34 (4.01%)
Philips Epiq7	9 (1.06%)	17 (2.00%)	26 (3.07%)
Sonoscape S40	18 (2.12%)	5 (0.59%)	23 (2.71%)
Mindray DC6	15 (1.77%)	5 (0.59%)	20 (2.36%)
GE Vivid7	12 (1.42%)	8 (0.94%)	20 (2.36%)
Aixplorer	6 (0.71%)	13 (1.53%)	19 (2.24%)
Siemens Acuson s2000	11 (1.30%)	7 (0.83%)	18 (2.12%)
Philips HD15	11 (1.30%)	3 (0.35%)	14 (1.65%)
Philips IE33	5 (0.59%)	7 (0.83%)	12 (1.42%)
Mindray DC8EXP	7 (0.83%)	3 (0.35%)	10 (1.18%)
GE Voluson E6	3 (0.35%)	7 (0.83%)	10 (1.18%)
Mindray DC6T	7 (0.83%)	2 (0.24%)	9 (1.06%)
Esaote Mylab60	3 (0.35%)	4 (0.47%)	7 (0.83%)
Esaote Mylab7	5 (0.59%)	1 (0.12%)	6 (0.71%)
GE Voluson E8	2 (0.24%)	3 (0.35%)	5 (0.59%)
Mindray 8PRO	0 (0.0%)	3 (0.35%)	3 (0.35%)
Sonoscape SSI6000	1 (0.12%)	0 (0.0%)	1 (0.12%)
Mindray M9	1 (0.12%)	0 (0.0%)	1 (0.12%)
Mindray DC8	1 (0.12%)	0 (0.0%)	1 (0.12%)

### 3.2 Comparison among the U‐net and the Attention U‐net of classification performance

**FIGURE 3:**
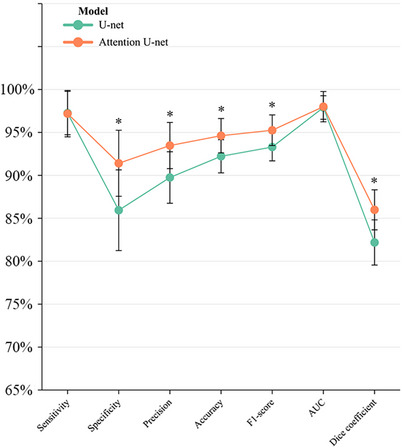
The sensitivity, specificity, precision, accuracy, F1‐score, AUC, and average dice coefficient for detection and segmentation by the U‐net and the Attention U‐net. The Attention U‐net performed better at detecting and segmenting the image of pleural effusion than the U‐net. AUC, area under the cure.^*^The data were statistically different between the two groups (*p* < 0.05).

Figure 3 and Table S1 compares the experimental results of different models for pleural effusion detection under identical conditions. The Attention U‐net in this study achieves better results in evaluating classification performance indicators. Figure 3 lists the sensitivity, specificity, precision, accuracy, F1‐score, and AUC for detection by the U‐net and the Attention U‐net. The index of specificity, precision, accuracy, and F1‐score with the U‐net and the Attention U‐net was significantly distinct (*p* < 0.05).

**FIGURE 4:**
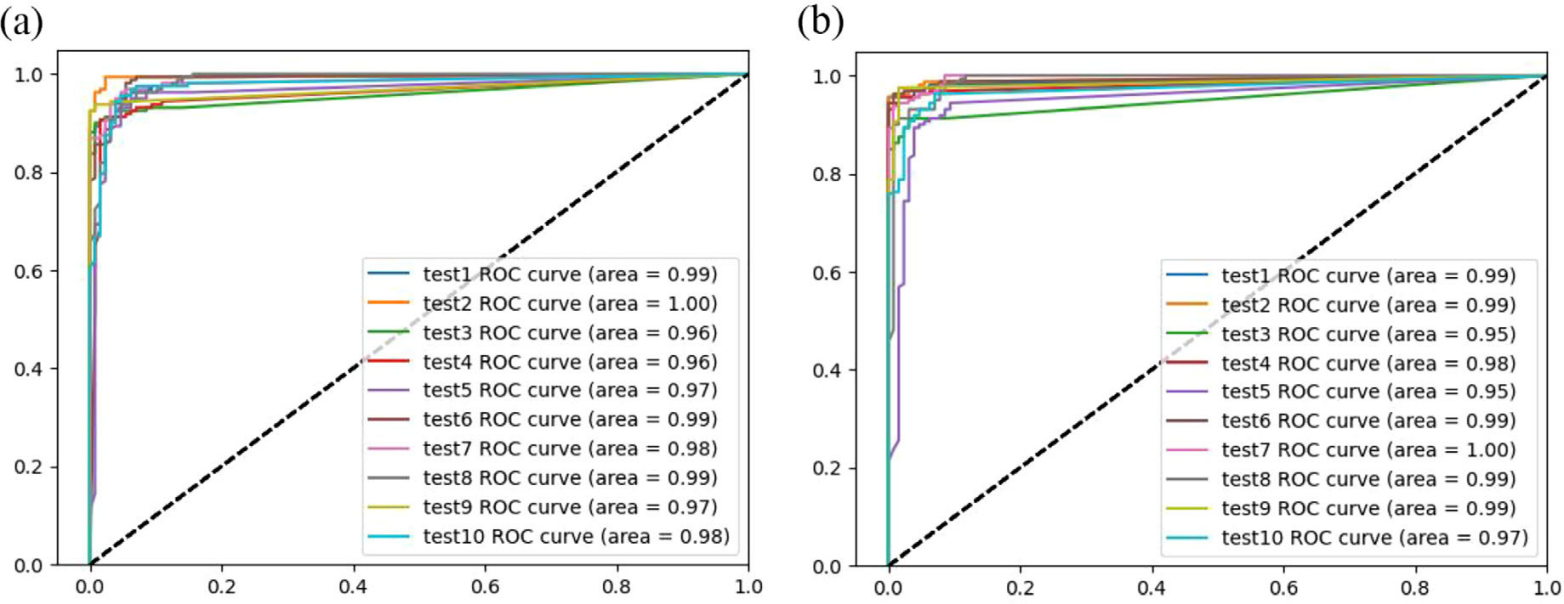
ROC curve for detecting pleural effusion, as the prediction results from the models. (a) and (b) are the results of ten experiments of the U‐net and the Attention U‐net respectively. The AUC for the U‐net model ranged from 0.96 to 1.0 and for the Attention U‐net from 0.95 to 1.0, demonstrating that the pleural effusion was well detectable. The low volatility of the 10 results shows that the models trained by our data have good stability in diagnosing pleural effusion. AUC, area under the cure; ROC, receiver operating characteristic.

The two models had no significant change in the sensitivity and AUC of pleural effusion detection. The sensitivity ranged from 93% to 100% by the U‐net model and 91%to 100% by the Attention U‐net in ten tests. As shown in Figure 4, in 10 tests, the AUC for the U‐net model ranged from 0.96 to 1.0 and for the Attention U‐net from 0.95 to 1.0.

The specificity ranged from 77% to 94% by the U‐net and 84% to 98% by the Attention U‐net in 10 tests. The specificity was greatly optimized in the Attention U‐net (*p* = 0.012). The two models' precision ranged from 85% to 95% and 89% to 99%, respectively (*p* = 0.013). The accuracy varied between 90% and 95% and 91% and 98%, respectively (*p* = 0.020). The F1‐score ranged from 91% to 96% and 92% to 98% in the two models, respectively (*p* = 0.027).

### 3.3 Comparison among the U‐net and the Attention U‐net of segmentation performance

**FIGURE 5:**
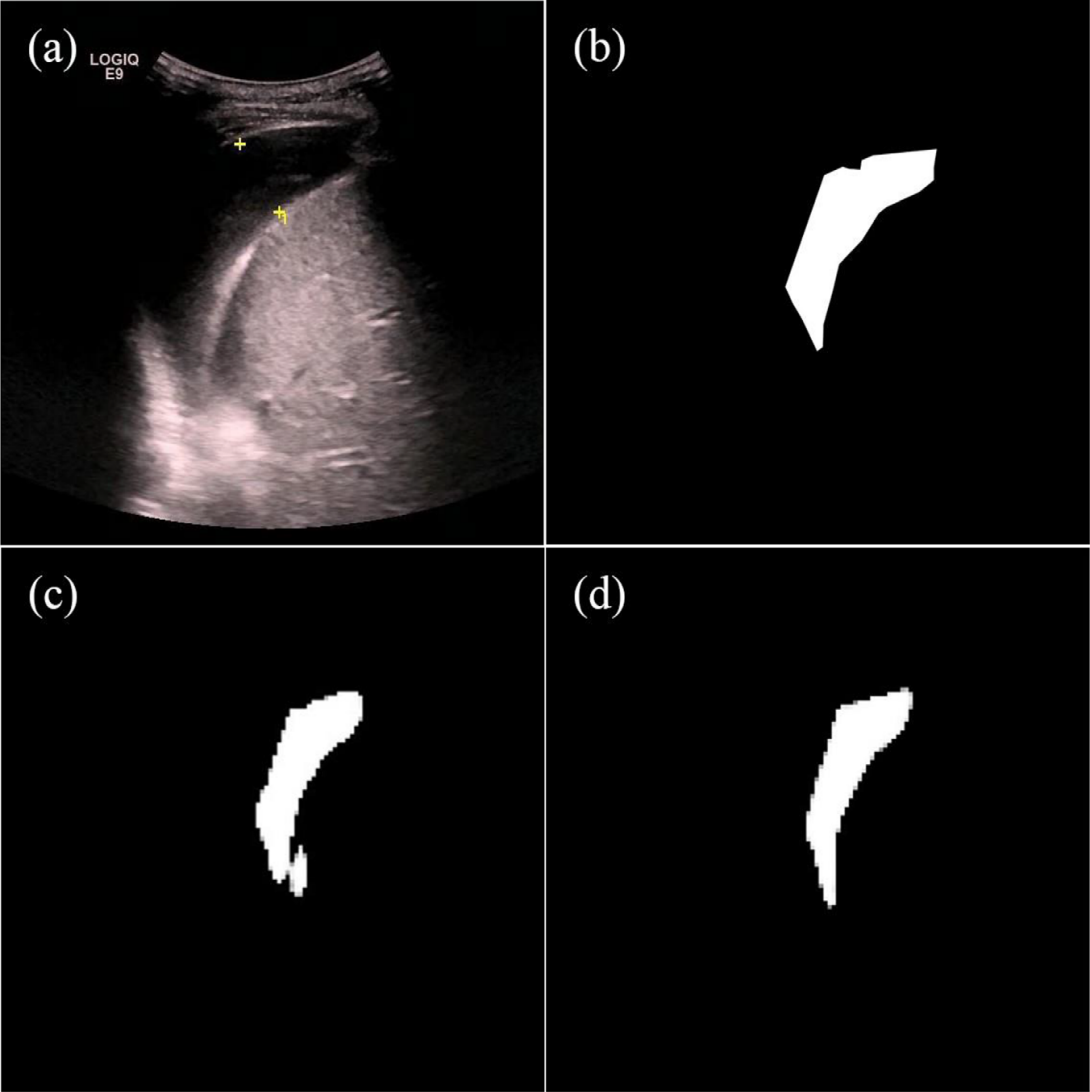
Typical target area contours achieved automatically by the U‐net and the Attention U‐net. (a) A sample image was collected from PACS. (b) The image after annotation by manual. (c) and (d) The ROIs automatically outlined by the U‐net and the Attention U‐net, respectively. The U‐net dice coefficient is 0.87 and the Attention U‐net is 0.93. PACS, Picture Archiving and Communication Systems; ROIs, regions of interest.

Figure 5 presents typical target area contours achieved by the U‐net and the Attention U‐net automatic segmentation models and their comparison with manual contours on US images for pleural effusion, respectively. The segmentation effect of the Attention U‐net in this study is significantly better than the U‐net in terms of overall and detail processing. Figure 3 shows different models for pleural effusion segmentation under identical conditions. In 10 tests, the average dice coefficient varied between 0.79 and 0.86 by the U‐net and 0.83 and 0.90 by the Attention U‐net. The segmentation performance has been markedly increased (*p* = 0.003), which indicates that the attention U‐net has better segmentation performance.

## 4 DISCUSSION

In this study, we employed the Attention U‐net, an improved version of the U‐net, to automatically detect and segment the ultrasound images of pleural effusion. The model was trained on 5184 photos and subsequently tested on 576 images. The Attention U‐net exhibited an average accuracy of 94%, an AUC of 0.98 and an average dice coefficient of 0.86, highlighting the potential of deep learning models in diagnosing pleural effusion on ultrasound images.

Our deep learning model is optimized for data sets to improve its usefulness in emergency situations. Prior studies have demonstrated that pleural effusion ultrasound images are amenable to machine learning. Tsai et al.^23^ utilized a Reg‐STN model to identify pleural effusion in a binary method in 2021, achieving an average accuracy of 92%, which is consistent with our study's U‐net‐based results for pleural effusion classification (92%). However, the Attention U‐net demonstrated higher categorization accuracy (95%) in this study. Meanwhile, their image acquisition was standardized, with the device and scanning location being controlled, which is difficult to achieve in practical clinical settings, particularly in emergency situations. Routine chest scans are typically conducted along the anterior axillary line, midaxillary line, and posterior axillary line. However, practical limitations such as patient positioning and other factors may sometimes necessitate deviations from the standard scanning positions. To address this issue, our study did not standardize the device or scanning position, instead focusing on the other suitable parameters for rapid diagnosis in emergency scenarios. Moreover, Tsai et al. utilized weakly supervised learning to study ultrasound images of 70 patients. Given the poor image quality and the interpretation challenge, ensuring the ground truth's accuracy is critical to establishing the validity of test datasets for deep learning.^24^ To obtain the most precise ground truth possible, our study employed fully supervised learning, with label images manually outlined by two experienced sonographers for 848 subjects. Segmentation of the target area is a critical and necessary step for quantitative analyses in clinical practice.^25^ In addition to pleural effusion classification, our program generates contours of the effusion. It produces outcomes with an average dice coefficient of 0.82 for the U‐net and 0.86 for the Attention U‐net. Once the program is trained to automatically outline the area of pleural effusion, physicians without ultrasound training can perform ultrasound‐guided punctures for pleural effusion, substantially reducing puncture‐related complications such as pneumothorax, which can occur at a rate of 9.3% without ultrasound guidance and 4% with it.^25^ One of the primary objectives of this study was to develop a model that could assist non‐sonographers in conducting ultrasound guidance while performing a thoracopuncture.

The experimental results of the pleural effusion dataset showed that the Attention U‐net outperformed the U‐net in detection and segmentation. Both models had an average sensitivity of 97%, indicating that pleural effusion was well detectable. Further, the Attention U‐net had a higher average specificity of 91% compared to 86% for the U‐net, which meaningfully reduced false positives (from 14% to 9%). Reducing false positives is essential because they could result in inappropriate clinical interventions, such as urgent surgery. The study has shown that the attention mechanism can transform the original image's spatial information into another space while retaining essential information or properties.^26^ In other words, adding AGs to the U‐net can improve the feature extraction ability of the model. In the ultrasonic image of the pleural cavity, negative images without pleural effusion often show acoustic shadows behind the ribs and the gas, which may resemble pleural effusion. The Attention U‐net model clearly outperformed the U‐net in recognizing acoustic shadows. The Attention U‐net results' accuracy, precision, and F1 score were better than those of the U‐net. The two models were put through ten‐fold tests, as shown in Figure 4, and the ROC curve revealed that the models had excellent stability in the diagnosis of pleural effusion in this study. Therefore, the models developed using our dataset can more accurately represent the various conditions that may occur in clinical practice. Additionally, the Attention U‐net was more accurate in predicting the pleural effusion region, as indicated by its average dice coefficient of 0.86, compared to the U‐net's average dice coefficient of 0.82. Figure 5 shows that the Attention U‐net successfully decreased over‐segmentation and under‐segmentation while achieving smoother edges. This is crucial for future studies since it will allow the model to estimate the effusion size more accurately and direct doctors during thoracic punctures.

When it comes to identifying pleural effusions, AI diagnostic models based on radiological images have been previously developed.^24^, ^27^, ^28^, ^29^ For instance, Niehues et al.^24^ demonstrated that deep learning improved the diagnosis of pleural effusion in intensive care and emergency medicine, utilizing bedside chest radiographs and achieving an AUC of 0.85. While chest radiographs and CT images are commonly used, ultrasound‐based detection of pleural effusion offers the advantages of real‐time imaging, absence of ionizing radiation, and portability, making it highly suitable for emergency trauma situations. Previous literature have shown that thoracic ultrasound is more accurate than x‐ray and as accurate as CT in detecting effusion.^30^ However, the number of studies focusing on models based on ultrasound images is limited. Thus, our research contributes additional insights into AI diagnostic models for pleural effusions. In addition, while many researchers have incorporated evaluation indicators for ascites and pericardial effusion into AI research within E‐FAST tests over the past 2 years,^8^, ^12^, ^13^, ^14^, ^15^ there have been relatively few studies focusing on pleural effusion. Our research serves as a valuable complement to the application of E‐FAST indicators in the field of AI.

The automatic segmentation model for pleural effusion not only aids in the execution of E‐FAST but also holds the potential to be beneficial in various other application scenarios. Firstly, it can be applied to critical patients in the ICU to provide clinicians with real‐time detection of pleural effusion, thus improving the diagnostic ability of FAFF.^31^ Secondly, it can enable physicians without ultrasound training to perform ultrasound‐guided punctures for pleural effusion, reducing the complications associated with punctures.^32^ Finally, the model can help senior sonographers complete medical instruction and quality assurance work in the process. In summary, the model's potential to improve the detection and segmentation of pleural effusion can have a positive impact on diagnostic capabilities, patient care, and reduce the complications of pleural puncture.

The proposed method has some limitations that need to be addressed in future studies. Firstly, this study only produced the image's contour, and the dataset failed to estimate fluid accumulation. Moreover, our study is from single‐center studies. To make the deep learning model developed in this study generally applicable, large‐scale external validations from multiple centers are required in the future. Additionally, the model should be continuously improved by adding new datasets from different devices, parameters, populations, etc., to train the model and improve its diagnosis performance continuously.

## 5 CONCLUSION

The Attention U‐net model exhibits high efficiency and accuracy in detecting and segmenting pleural effusion on ultrasound images. The findings suggest that it is feasible and reliable to develop software placement relevant to portable ultrasonic devices, which can assist novice physicians, clinicians without ultrasonic imaging expertise, or non‐professional individuals in diagnosing pleural effusion rapidly and accurately using ultrasound images. This technology has the potential to enhance the operation and application of E‐FAST in clinical work.

## AUTHOR CONTRIBUTIONS

*Conception and design*: Libing Huang, Zhengyi Li; *Administrative support*: Zhengyi Li; *Provision of study materials or patients*: Yingying Lin, Peng Cao, Qian Qin, Libing Huang; *Collection and assembly of data*: Fengting Liang, Zhanye Lin, Libing Huang; *Data analysis and interpretation*: Libing Huang, Xia Zou, Zhengyi Li; *Manuscript writing*: All authors; *Final approval of manuscript*: All authors.

## CONFLICT OF INTEREST STATEMENT

The authors declare no conflicts of interest.

## ETHICAL APPROVAL

This study was approved by the Ethics Committee of Shenzhen Second People's Hospital.

## CONSENT TO PARTICIPATE

The ethics committee of Shenzhen Second People's Hospital (approval number 20220324001) waived the informed consent due to the retrospective observational nature of the study.

## Supporting information

Supporting information

## Data Availability

The data supporting this study's findings are not publicly available as the information contained could compromise the privacy of research participants. Further inquiries can be directed to the corresponding author.
