# Supplementary material for: Automated detection and segmentation of pleural effusion on ultrasound images using an Attention U‐net
Source: J Appl Clin Med Phys. 2023 Dec 13;25(1):e14231. doi: 10.1002/acm2.14231 (PMC10795456; doi:10.1002/acm2.14231)
Supplement: Supplementary file 1 — Supporting information [file ACM2-25-e14231-s001.docx]

|  | **Test 1** | **Test 2** | **Test 3** | **Test 4** | **Test 5** | **Test 6** | **Test 7** | **Test 8** | **Test 9** | **Test 10** |
| --- | --- | --- | --- | --- | --- | --- | --- | --- | --- | --- |
| **Sensitivity** |  |  |  |  |  |  |  |  |  |  |
| U-net | 99.38% | 99.38% | 93.13% | 94.38% | 96.25% | 99.38% | 98.13% | 100.00% | 94.38% | 98.13% |
| Attention U-net | 98.13% | 98.75% | 91.25% | 96.88% | 94.38% | 98.75% | 100.00% | 100.00% | 97.50% | 96.25% |
| **Specificity** |  |  |  |  |  |  |  |  |  |  |
| U-net | 84.38% | 89.06% | 85.94% | 89.06% | 84.38% | 89.84% | 84.38% | 77.34% | 93.75% | 81.25% |
| Attention U-net | 93.75% | 93.75% | 91.41% | 91.41% | 90.63% | 92.19% | 84.38% | 86.72% | 98.44% | 91.41% |
| **Precision** |  |  |  |  |  |  |  |  |  |  |
| U-net | 88.83% | 91.91% | 89.22% | 91.52% | 88.51% | 92.44% | 88.70% | 84.66% | 94.97% | 86.74% |
| Attention U-net | 95.15% | 95.18% | 92.99% | 93.37% | 92.64% | 94.05% | 88.89% | 90.40% | 98.73% | 93.33% |
| **Accuracy** |  |  |  |  |  |  |  |  |  |  |
| U-net | 92.71% | 94.79% | 89.93% | 92.01% | 90.97% | 95.14% | 92.01% | 89.93% | 94.10% | 90.63% |
| Attention U-net | 96.18% | 96.53% | 91.32% | 94.44% | 92.71% | 95.83% | 93.06% | 94.10% | 97.92% | 94.10% |
| **F1-score** |  |  |  |  |  |  |  |  |  |  |
| U-net | 93.81% | 95.50% | 91.13% | 92.92% | 92.22% | 95.78% | 93.18% | 91.69% | 94.67% | 92.08% |
| Attention U-net | 96.62% | 96.93% | 92.11% | 95.09% | 93.50% | 96.34% | 94.12% | 94.96% | 98.11% | 94.77% |
| **AUC** |  |  |  |  |  |  |  |  |  |  |
| U-net | 99.00% | 100.00% | 96.00% | 96.00% | 97.00% | 99.00% | 98.00% | 99.00% | 97.00% | 98.00% |
| Attention U-net | 99.00% | 99.00% | 95.00% | 98.00% | 95.00% | 99.00% | 100.00% | 99.00% | 99.00% | 97.00% |
| **Average dice** |  |  |  |  |  |  |  |  |  |  |
| U-net | 81.36% | 85.52% | 80.23% | 82.42% | 81.79% | 84.95% | 80.67% | 79.21% | 86.51% | 79.17% |
| Attention U-net | 86.66% | 88.83% | 84.54% | 86.58% | 84.28% | 87.69% | 82.53% | 83.80% | 89.82% | 85.12% |
